# DUSP4 Silencing Enhances the Sensitivity of Breast Cancer Cells to Doxorubicin through the Activation of the JNK/c-Jun Signalling Pathway

**DOI:** 10.3390/molecules27196146

**Published:** 2022-09-20

**Authors:** Mashael S. Al-Mutairi, Hany O. Habashy

**Affiliations:** 1 Department of Medical Laboratory Sciences, Faculty of Allied Health Sciences, Health Science Center, Kuwait University, Safat P.O. Box 5969, Kuwait; 2 Department of Histopathology and Cytology, Al-Adan Hospital, Ministry of Health, Kuwait, and Faculty of Medicine, Mansoura University, Mansoura 35516, Egypt

**Keywords:** chemoresistance, doxorubicin, DUSP4, EMT, JNK, breast cancer

## Abstract

Doxorubicin (Dox) has limited efficiency in breast cancer (BC) due to drug-acquired resistance. The epithelial–mesenchymal transition (EMT) plays a major role in the survival and drug resistance of cancer cells. It was suggested that the JNK pathway was implicated in the response to Dox by regulating EMT. DUSP4/or MKP-2 is a well-known regulator of the JNK pathway and was found to be highly expressed in BC. However, its functional significance is not yet fully understood. In the present study, the possible involvement of MKP-2 in Dox-induced EMT was investigated in breast cancer cells. Immunohistochemistry for tissues obtained from BC patients (*n* = 108) revealed 71.3% of tissues stained positively for MKP-2 while only 28.7% stained negatively. However, MKP-2 protein expression exhibited no significant relationship between BC prognostic factors, such as histological grade, histological type, hormonal status, and Ki-67 marker, its expression was significantly correlated with age 40 or below. In MDA-MB-231 cells, Dox-induced phosphorylation of JNK was sufficiently enhanced in MKP-2 silenced cells. This resulted in the attenuation of Dox-induced EMT, cell cycle arrest, and ultimately accelerated apoptosis. It was confirmed that the acquisition of Dox sensitivity by MKP-2 silencing largely depends on the stimulation of the JNK pathway. Indeed, results showed that overexpressing MKP-2 in non-tumorigenic MCF-12A cells dramatically inhibited Dox-induced JNK activation and, subsequently, cell death. The present study, to our knowledge, is the first to provide evidence for the potential role of MKP-2 in chemoresistance to Dox through modulating the JNK pathway and enhancing EMT.

## 1. Introduction

Breast cancer (BC) has the highest frequency and occurrence among all cancers affecting women worldwide and is considered the second leading cause of cancer-related deaths in women [1]. Resistance, recurrence, and multiple side effects remain frequent despite the clear improvement in the survival of BC patients treated with conventional therapies, such as surgery, radiotherapy, chemotherapy, and other modern treatments [1]. Doxorubicin (Dox), an anthracycline chemotherapeutic agent, along with others from the same group, is important for the treatment of breast cancer. The epithelial-to-mesenchymal transition (EMT) process plays major roles in cancer progression, poor disease prognosis, and resistance to therapy [2,3]. Accumulating evidence has shown that the repeated development of Dox resistance in cancer may be linked to EMT [4,5,6], and many factors involved in EMT-mediated Dox resistance have been established [7]. However, the exact mechanisms by which EMT is linked to Dox resistance remain unknown. Multiple signalling pathways linked to EMT play a role in the development of chemoresistance [8]. Several studies have implicated c-Jun N-terminal kinase (JNK) as an important activator of EMT and EMT-associated phenotypes [9,10]. JNK deletion reverses EMT in mouse embryonic fibroblasts. This was determined by the increase in E-cadherin, decrease in N-cadherin, and colony-forming ability [11]. Studies have also demonstrated that JNK can promote tumor development in many cancers, including human BC [12,13]. Evidence indicates that JNK regulates cancer cell apoptosis and survival [14]. JNK activation plays a crucial role in Dox-induced cell death in cancer cells [15]. Thus, the regulation of JNK activity may be involved in the chemoresistance of breast cancer cells to Dox.

Mitogen-activated protein kinase phosphatase-2 (MKP-2), also known as DUSP4, belongs to a subgroup of dual-specificity phosphatases (DUSPs). MKP-2 plays a crucial role in the regulation of mitogen-activated protein kinase (MAPK) signalling pathways [16]. Although MKP-2 has been linked to a variety of cancer types, there are conflicting data on the involvement of MKP-2 as a negative regulator of the MAPK signalling pathway. A number of studies have associated down-regulation of MKP-2 with the progression of several types of cancer, including BC [17,18], pancreatic cancer [19], and colorectal cancer [20]. Others have indicated that MKP-2 overexpression promotes cancer development and progression [21,22,23,24,25]. In addition, several studies have reported that MKP-2 is linked to drug resistance in multiple cancer types [16,26]. MKP-2 suppression abrogates Dox-induced EMT, whereas its overexpression increases EMT [23]. Recently, MKP-2 was found to be directly connected to EMT/cancer stem cell (CSC)-inducible gene promoters [27]. However, the mechanisms by which the MKP-2/MAPK pathway regulates breast EMT and alters the cellular phenotype that promotes chemoresistance remain unclear. Therefore, we sought, in the present study, to examine the potential role of MKP-2-mediated EMT in Dox-dependent chemoresistance. Here, we show that MKP-2 silencing induces apoptosis and enhances Dox chemosensitivity by activating the JNK pathway. In contrast, MKP-2 overexpression in non-tumorigenic MCF-12A cells considerably inhibited Dox-induced JNK activation and subsequent cell death. The present results provide evidence for the potential role of MKP-2 in enhancing EMT and chemoresistance to Dox by modulating the JNK pathway.

## 2. Results

### 2.1. MKP-2 Expression and Correlation with Clinicopathological Breast Cancer Parameters

**Figure 1:**
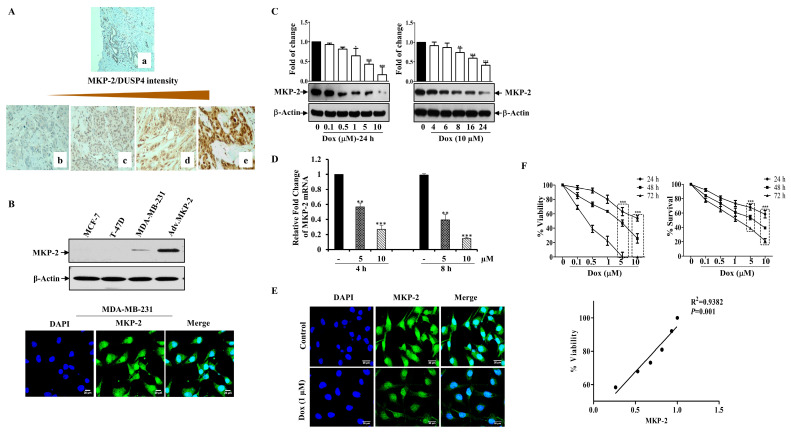
Dox Inhibits MKP-2 Expression and Reduces Cell Viability. (**A**) MKP-2 protein expression in breast carcinoma cases. (**a**) Normal breast tissue with negative MKP-2 expression, ×100. (**b**) Invasive ductal carcinoma with negative MKP-2 expression, ×200. (**c**) Invasive ductal carcinoma with mild MKP-2 expression, ×200. (**d**) Invasive ductal carcinoma with moderate MKP-2 expression, ×200. (**e**) Invasive ductal carcinoma with high MKP-2 expression, ×400. (**B**) MCF-7, T-47D, and MDA-MB-231 cell lysates were prepared, separated by SDS-PAGE, and then assessed for MKP-2 and β-Actin expression, and a confocal image of the immunofluorescence staining for MKP-2 in MDA-MB-231 cells was taken. (**C**) MDA-MB-231 cells were treated with increasing concentrations of Dox (0.1–10 µM) for 24 h or with 10 µM up to 24 h, then assessed for MKP-2 and β-Actin. (**D**) MDA-MB-231 cells were treated with Dox (5 or 10 µM) for 4 or 8 h, and then the expression of MKP-2 mRNA was analyzed by quantitative real-time PCR. (**E**) Immunofluorescent images of MKP-2 expression in control and cells treated with Dox (1 µM) for 24 h. (**F**) MDA-MB-231 cells were treated with increasing concentrations of Dox (0.1–10 µM) for 24, 48, or 72 h and then cell viability and % of surviving cells were assessed. A correlation analysis of MKP-2 protein expression and cell viability was calculated. Each value represents the mean ± S.E.M of three independent experiments. * *p* ≤ 0.05, ** *p* ≤ 0.01, *** *p* ≤ 0.001, compared with the untreated control.

In this study, we collected multiple BC specimens to investigate the clinical relevance of MKP-2 levels in BC disease progression. MKP-2 expression was determined using immunohistochemistry (Figure 1A), and the association between its expression and various histopathological parameters is shown in Table 1. MKP-2 protein expression levels were higher in BC tissues than in the adjacent non-tumorous tissues. Furthermore, 71.3% of all BC tissues were positive for MKP-2, whereas only 28.7% were negative for MKP-2 (Table 1). To investigate the link between MKP-2 protein level and clinical characteristics, samples were divided into low, moderate, and high-level groups according to immunohistochemical evaluation. Additionally, as shown in Table 1, MKP-2 expression exhibited no significant relationship between BC prognostic factors, such as histological grade (*p* = 0.977), histological type (*p* = 0.928), HER2 status (*p* = 0.804), PR (*p* = 0.696), and ER (*p* = 0.499). In addition, tumor size (*p* = 0.446) and Ki-67 values (*p* = 0.469) were both found not to be associated with MKP-2 expression. However, MKP-2 showed significant staining in samples from patients aged ≤40 years (*p* = 0.04). 

### 2.2. Dox Inhibits MKP-2 Expression and Breast Cancer Cell Growth

Based on these results, we sought to study the impact of MKP-2 on BC chemotherapy. To this end, we first assessed the basal MKP-2 protein levels in three different BC cell lines (MDA-MB-231, MCF-7, and T-47D) by Western blotting. Figure 1B shows the high MKP-2 protein levels in MDA-MB-231 cells when MKP-2 was ectopically expressed as a positive control (adenovirus). Interestingly, while MKP-2 was undetectable in the luminal BC cells MCF-7 and T47D, it was expressed in the triple negative BC cells MDA-MB-231 (Figure 1B). Consistently, immunofluorescence assays confirmed that MDA-MB-231 cells expressed a significant quantity of MKP-2 in their nuclei (Figure 1B). Therefore, MDA-MB-231 cells were used for subsequent experiments as they presented the highest MKP-2 expression level relative to the other cell lines. Next, we evaluated the effects of Dox on MKP-2 expression in MDA-MB-231 cells. MKP-2 protein expression significantly decreased in response to Dox when compared to basal values in a time- and concentration-dependent manner (Figure 1C). A similar effect was observed at the mRNA level (Figure 1D). Additionally, immunofluorescence staining showed a reduction in the MKP-2 nuclear staining intensity after Dox treatment (Figure 1E). Interestingly, Dox-dependent MKP-2 downregulation was accompanied by a dramatic reduction in cellular viability, as increasing concentrations of Dox significantly increased cell death in time- and concentration-dependent manners (Figure 1F). This finding was supported by the cell proliferation results. When we analyzed the relationship between MKP-2 downregulation and cell viability reduction in response to Dox treatment, the results showed a positive correlation (Figure 1F). These data suggest that Dox reduces the cell viability of MDA-231-MB cells, possibly through the downregulation of MKP-2. 

### 2.3. Dox Induces Apoptosis through the Mitochondrial Pathway and Arrests the Cell Cycle at G2/M

**Figure 2:**
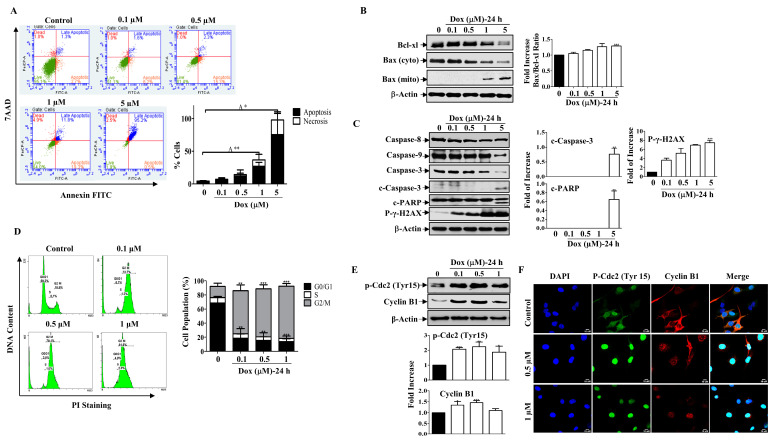
Dox Induces Mitochondrial Apoptosis and Cell Cycle Arrest. (**A**) MDA-MB-231 cells were treated with Dox concentrations (0.1–5 µM) for 24 h then apoptosis was detected by labelling cells with Annexin V-tagged FITC/7AAD and analyzed by flowcytometry. Lower right quadrant Annexin V-positive/7AAD-negative cells, denoted as early apoptotic cells, Annexin V-positive/7AAD-positive cells in the upper right quadrant, denoted as late apoptotic cells, and 7AAD-positive/Annexin V-negative cells in the upper left quadrant denote necrotic cells. The graph represents the % of apoptosis and necrosis for three independent experiments. Cells were treated with increasing concentrations of Dox (0.1–5 µM) for 24 h, then cell lysates were prepared, separated by SDS PAGE, and assessed for (**B**) Bcl-xl, Bax (cyto and mito), and β-Actin. (**C**) Caspase-8, Caspase-9, Caspase-3, c-Caspase-3, c-PARP, p-γ-H2AX, and β-Actin. (**D**) Cells were analyzed for cell cycle using PI-staining. The graph represents the % of cells in each cell cycle phase. (**E**) Cyclin B1 and p-Cdc2 (Tyr15) protein expressions were measured. (**F**) Immunofluorescent staining for both proteins were assessed. Each value represents the mean ± S.E.M of three independent experiments. * *p* ≤ 0.05, ** *p* ≤ 0.01, *** *p* ≤ 0.001 compared with the correspondent control.

Next, we examined the mode of Dox-mediated cell death in MDA-MB-231 cells. Annexin V/PI-associated flow cytometric analysis indicated that cells entered the apoptotic stage 24 h after treatment with increasing concentrations of Dox, and only a small population of cells became necrotic when compared to untreated cells (Figure 2A). Consistent with this finding, Dox significantly downregulated the levels of the pro-survival protein Bcl-xl and the cytosolic pro-apoptotic protein Bax in a concentration-dependent manner. Additionally, there was a gradual increase in Bax protein expression in the mitochondria. The ratio of Bax to Bcl-xl proteins increased in a concentration-dependent manner (Figure 2B). As a result, gradual degradation of Caspase-9 was detected, which subsequently caused degradation of the Caspase-3 protein. This led to the appearance of cleaved Caspase-3, which peaked significantly at high Dox concentrations and stimulated PARP cleavage, a downstream substrate of Caspase-3 (Figure 2C). In contrast, there was no detectable change in the Caspase-8 protein level in response to Dox treatment (Figure 2D). Moreover, the level of phosphorylated γ-H2AX increased upon treatment with Dox in a concentration-dependent manner (Figure 2C). γ-H2AX is a marker of DNA damage, which is considered a molecular event associated with apoptosis and cell cycle arrest [28]. This indicated that Dox promoted apoptosis through the mitochondrial pathway in BC cells.

Furthermore, treating cells with low concentrations of Dox resulted in an accumulation of cells in the G2/M phase after 24 h of treatment compared to control cells (Figure 2D). To further understand the mechanism underlying Dox-induced G2/M cell cycle arrest, we examined the expression of cell cycle regulatory proteins for the G2/M transition, such as cyclin B1 and p-Cdc2 (Tyr15), by Western blotting. Figure 2E shows that Dox treatment for 24 h increased the phosphorylation of the inhibitory kinase Cdc2 (Tyr 15), which was accompanied by an increase in the level of Cyclin B1 (Figure 2E). The immunofluorescent staining results support the immunoblot analysis, as cyclin B1 and p-Cdc2 showed nuclear translocation after Dox treatment compared to the control (Figure 2F). In contrast, in the untreated controls, both proteins were mostly localized in the cytoplasm, and only a small amount was present in the nucleus. These results suggest that Dox induces G2/M cell cycle arrest through activation of Cdc2 and cyclin B1 proteins.

### 2.4. Downregulation of MKP-2 Enhances the Sensitivity of MDA-MB-231 Cells to Dox

**Figure 3:**
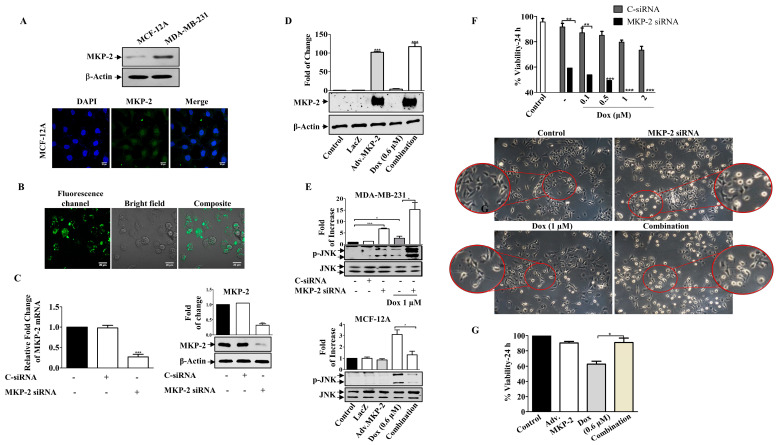
MKP-2 Silencing Induced Breast Cancer Cell Sensitivity to Dox. (**A**) MCF-12A and MDA-MB-231 cell lysates were prepared, separated by SDS-PAGE, and then assessed for MKP-2. In addition, a confocal image of immunofluorescence staining for MKP-2 in MCF-12A cells was taken. MDA-MB-231 cells were transfected with MKP-2 siRNA (10 nm) for 72 h then (**B**) the efficiency was assessed by measuring fluorescence using an MKP-2 siRNA fluorescent negative control and (**C**) by detecting MKP-2 mRNA and protein levels using qRT-PCR and Western blot. (**D**) MCF-12A cells were infected with Adv. MKP-2 (500 pfu/cell) for 24 h, then cell lysates were prepared and assessed for MKP-2 and β-Actin. After transfection or infection with MKP-2, MDA-MB-231, and MCF-12A, cells were treated with Dox (0.6 or 1 µM) for 24 h, and then cell lysates were prepared and assessed for (**E**) *p*-JNK and JNK, and (**F**,**G**) cell viability. (**F**) morphological changes were detected using phase contrast microscopy (×20). Each value represents the mean ± S.E.M of three independent experiments. * *p* ≤ 0.05, ** *p* ≤ 0.01, *** *p* ≤ 0.001 compared with the correspondent control.

To explore the functional significance of MKP-2, we silenced the gene in MDA-MB-231 cells or overexpressed the MKP-2 gene (adenoviral) in non-tumorigenic MCF-12A BC cells (Figure 3). MCF-12A cells expressed low levels of MKP-2 protein compared to MDA-MB-231 cells, which was confirmed by Western blot and immunostaining assays (Figure 3A). The silencing efficacy of siRNA was confirmed by using a fluorescent negative control, which showed positive staining (Figure 3B), and by measuring both MKP-2 mRNA and protein levels (Figure 3C). Similarly, ectopic expression of MKP-2 (Adv.MKP-2) in MCF-12A cells was confirmed by Western blotting (Figure 3D). To further verify the success of silencing or overexpressing MKP-2, the levels of JNK activation were tested in both cell lines (Figure 3E). As siRNAs sufficiently suppressed the expression of MKP-2, it also resulted in a significant induction of JNK phosphorylation levels after Dox treatment when compared to Dox alone (*p* ≤ 0.05). In contrast, Adv. MKP-2 significantly reduced Dox (IC_50_: 0.6 μM)-induced JNK phosphorylation (*p* ≤ 0.01). These results indicate the role of MKP-2 in cell sensitivity to Dox treatment. To further explore this, we examined cell viability in response to Dox. MKP-2 siRNA increased cell sensitivity to the cytotoxic effect of Dox as viability was dramatically reduced with increasing concentrations of Dox (Figure 3F). In addition, MKP-2 siRNA in untreated cells significantly decreased cell viability compared to that of cells transfected with C-siRNA (*p* ≤ 0.01). Consistent with these findings, cells treated with MKP-2 siRNA presented an obvious morphological change (Figure 3F) from the spindle phenotype (mesenchymal phenotype) to a rounded or cobblestone phenotype (epithelial phenotype), which increased in the presence of Dox. On the other hand, the inhibition of cell viability by Dox was reversed in the presence of Adv. MKP-2 (*p* ≤ 0.05) when compared to cells treated with Dox alone (Figure 3G). These results suggest that MKP-2 protects MDA-MB-231 cells from Dox-induced cell death and may therefore play an important role in chemoresistance.

### 2.5. Dox-Induced Cell Cycle Arrest and Apoptosis Is MKP-2-Dependent

**Figure 4:**
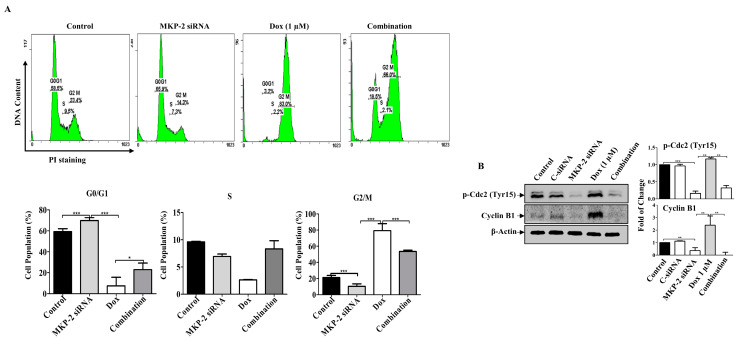
MKP-2 Silencing Promotes Cell Cycle Arrest in Breast Cancer Cells. MDA-MB-231 cells were transfected with MKP-2 siRNA (10 nM) for 72 h and then treated with the indicated concentrations of Dox for 24 h. (**A**) Representative cell cycle distribution was analyzed by flow cytometry. (**B**) Cell lysates were prepared, separated by SDS-PAGE and assessed for Cyclin B1 and p-Cdc2 (Tyr15). Each value represents the mean ± S.E.M of three independent experiments. * *p* ≤ 0.05, ** *p* ≤ 0.01, *** *p* ≤ 0.001, compared with the correspondent control.

**Figure 5:**
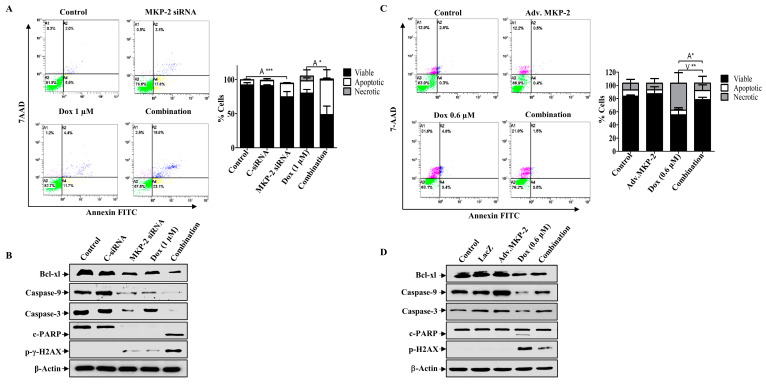
MKP-2 Silencing Promotes Cell Apoptosis in Breast Cancer Cells. MKP-2 was silenced from MDA-MB-231 cells or infected with Adv. MKP-2 (500 pfu/cell) for 24 h, then treated with indicated concentrations of Dox for 24 h, then (**A**,**C**) Apoptosis was detected by labelling cells with Annexin V-tagged FITC/7AAD and analyzing them by flowcytometry. (**B**,**D**) Cell lysates were prepared, separated by SDS-PAGE, and then assessed for Bcl-xl, Bax, Caspase-9, Caspase-3 c-Caspase-3, c-PARP, p-γ-H2AX, and β-Actin. Each value represents the mean ± S.E.M of three independent experiments. * *p* ≤ 0.05, ** *p* ≤ 0.01, *** *p* ≤ 0.001, compared with the correspondent control.

Next, we investigated the role of MKP-2 in Dox-related cell cycle arrest and apoptosis. MKP-2 silencing resulted in the accumulation of cells in the G0/G1 phase, whereas Dox-treated cells were arrested in the G2/M phase (Figure 4A). MKP-2 silencing significantly increased the proportion of cells in the G0/G1 phase and significantly reduced the proportion of cells arrested in the G2/M phase upon Dox treatment (Figure 4A). Accordingly, MKP-2 silencing caused a reduction in the levels of both p-Cdc2 and cyclin B1 compared to the controls or cells treated with Dox alone (Figure 4B). These results indicate that MKP-2 inhibited Dox-dependent cell cycle arrest at the G2/M phase. Furthermore, the impact of silencing or overexpressing MKP-2 on Dox-induced apoptosis was examined. MKP-2 knockdown significantly enhanced the number of cells undergoing apoptosis compared to the untreated control (*p* ≤ 0.001). This effect was remarkably increased when MKP-2 deficient cells were treated with Dox compared to those in control cells (*p* ≤ 0.05) (Figure 5A). At the molecular level, specific MKP-2 downregulation reduced the level of Bcl-xl protein compared to untreated cells (*p* ≤ 0.001). This effect was significantly enhanced upon treatment with Dox compared to control cells (*p* ≤ 0.01). As a result, downstream caspases were activated in the presence of MKP-2 siRNA alone and silencing enabled Dox to abolish Caspase-9 expression as compared to control cells (*p* ≤ 0.001) (Figure 5B). A similar effect was observed for Caspase-3 (*p* ≤ 0.01) and its downstream substrate, PARP (*p* ≤ 0.01) (Figure 5B). Consequently, MKP-2 siRNA induced DNA damage, as measured by the high phosphorylation of γ-H2AX, which increased significantly in the presence of Dox (*p* ≤ 0.05) compared to control cells. In contrast, ectopic MKP-2 expression significantly reduced apoptosis induced by Dox and protected MCF-12A cells from death (Figure 5C). Adv. MKP-2 upregulated the expression of Bcl-xl (*p* ≤ 0.01), and as a result, a significant increase in the levels of Caspase-9 (*p* ≤ 0.01) and Caspase-3 (*p* ≤ 0.05), and a reduction in c-PARP proteins (*p* ≤ 0.01) was detected after Dox treatment (Figure 5D). In addition, MKP-2 significantly protected cells from DNA damage induced by Dox compared to control cells treated with Dox alone (*p* ≤ 0.05). These results indicated that MKP-2 inhibited Dox-dependent induction of apoptosis. 

### 2.6. MKP-2 Reduces EMT Induced by Dox in MDA-MB-231 Cells

**Figure 6:**
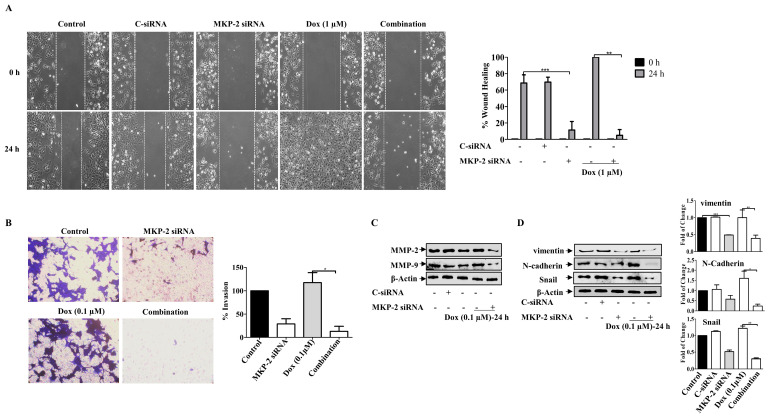
MKP~2 Promotes Dox Resistance Through EMT. MDA-MB-231 cells were transfected with MKP-2 siRNA for 72 h, then treated with the indicated Dox concentrations for another 24 h. (**A**) The wounded area was photographed (10×) and % of wound closure was evaluated and the result expressed as a percentage based on the ratio of the treated cells to the controls. (**B**) A cell invasion assay was performed, and the invaded cells were photographed. Cell lysates were prepared, separated by SDS-PAGE, and then assessed for (**C**) MMP-2 and MMP-9 (**D**) N-cadherin, vimentin, Snail and β-Actin. Each value represents the mean ± S.E.M of three independent experiments. * *p* ≤ 0.05, ** *p* ≤ 0.01, *** *p* ≤ 0.001, compared with the correspondent control.

We then investigated the roles of MKP-2 and Dox in the migration and invasion of BC cells. As expected, while MKP-2 deficient cells showed a reduced migration capacity, treatment of control cells with Dox enhanced their migratory ability (Figure 6A). Interestingly, treatment of MDA-MB-231 cells with Dox, following MKP-2 siRNA transfection, markedly abrogated cell migration (Figure 6A). These findings suggest the involvement of MKP-2 in the migration of BC cells and in the Dox-dependent induction of the migratory capacity of these cells. Similar results were obtained for the invasive capacity of the BC cells (Figure 6B). This was confirmed by showing that MKP-2 siRNA significantly reduced the levels of matrix metalloproteinase, MMP-2 and MMP-9, compared with control cells (*p* ≤ 0.05, *p* ≤ 0.05, respectively) or cells treated with Dox alone (*p* ≤ 0.01, and *p* ≤ 0.01, respectively) (Figure 6C). Further reduction was observed when MKP-2 silenced cells were treated with Dox (*p* ≤ 0.01 and *p* ≤ 0.01, respectively). These findings suggest that MKP-2 may play a role in the ETM of BC cells. To test this hypothesis, we examined the levels of EMT protein markers in MDA-MB-231 cells, and the results showed a decrease in the levels of the mesenchymal markers N-cadherin, vimentin, and Snail in MKP-2 deficient cells relative to the controls (Figure 6D). In contrast, Dox treatment upregulated N-cadherin (*p* ≤ 0.01), vimentin (*p* ≤ 0.01), and Snail (*p* ≤ 0.01) compared with the respective untreated cells. This effect was abolished when MKP-2 was knocked down (Figure 5D). These results suggest that Dox promoted EMT in an MKP-2-dependent manner. 

### 2.7. Doxorubicin Promotes EMT in Breast Cancer Cells through JNK Activation

**Figure 7:**
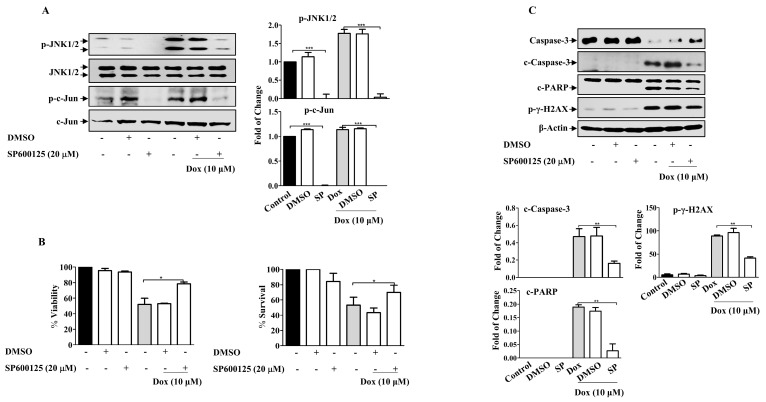
JNK Inhibition Attenuates Dox Cytotoxicity in Breast Cancer Cells. MDA-MB-231 cells were incubated with SP6000125 (20 µM) for 1 h, then treated with Dox (0.1,1 or 10 µM) for the indicated time. Cell lysates were prepared, separated by SDS-PAGE, and then assessed for (**A**) p-JNK, JNK, p-c-Jun, and c-Jun. (**B**) Cell viability was calculated and % of surviving cells was assessed using the MTT assay. (**C**) Caspase-3, c-Caspase-3, PARP, c-PARP, p-γ-H2AX, and β-Actin. Each value represents the mean ± S.E.M of three independent experiments. * *p* ≤ 0.05, ** *p* ≤ 0.01, *** *p* ≤ 0.001 compared with the correspondent control.

Given the role of MAPKs in cell survival and death, we sought to investigate the possible involvement of MAPKs in Dox-dependent effects. The present results revealed that Dox-activated JNK and its downstream protein c-Jun in a concentration- and time-dependent manner (Supplementary Figure S1), while no consistent effect on ERK and P38 MAPKs in MDA-MB-231 cells was detected (Supplementary Figure S2). We explored the involvement of JNK using SP600125, a JNK pharmacological inhibitor (Figure 7A). SP600125 significantly reduced the basal level of JNK compared to that in untreated control cells (*p* ≤ 0.001). Pre-incubation of cells with SP600125 before Dox treatment significantly attenuated JNK activation compared with cells treated with Dox alone (*p* ≤ 0.001). Furthermore, c-Jun phosphorylation was also inhibited (*p* ≤ 0.001). As expected, SP600125 significantly reversed the inhibitory effect of Dox on cell viability (*p* ≤ 0.05) (Figure 7B). This result was supported by the results of the MTT assay. Based on these results, we assessed the correlation between JNK activation and Dox-mediated apoptosis in MDA-MB-231 cells. SP600125 significantly reversed the effects of Dox-induced Caspase-3 (*p* ≤ 0.01) and PARP cleavage (*p* ≤ 0.01) (Figure 7C). This result correlated with the effect of SP600125 on γ-H2AX phosphorylation (*p* ≤ 0.01) (Figure 7C).

**Figure 8:**
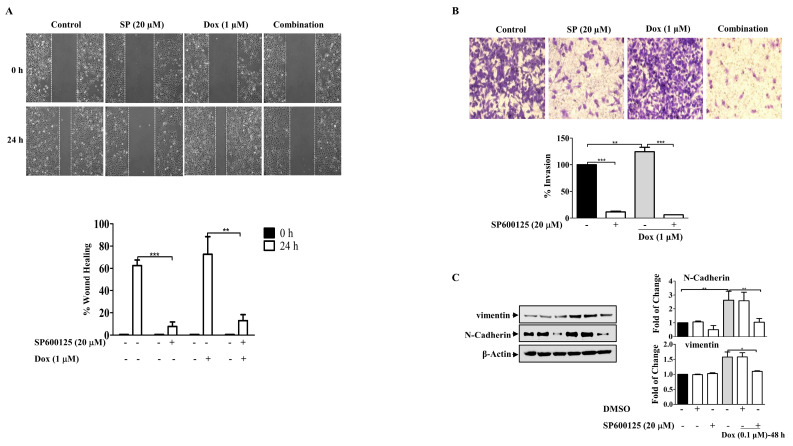
SP600125 Reverses the EMT-Induced By Dox In Breast Cancer Cells. MDA-MB-231 cells were incubated with SP6000125 (20 µM) for 1 h then treated with Dox (0.1 or 1 µM) for the indicated time. (**A**) The wounded area was photographed (10×) and % of wound closure was evaluated and the result expressed as a percentage based on the ratio of the treated cells to the controls. (**B**) A cell invasion assay was performed, and invaded cells were photographed. Cell lysates were prepared, separated by SDS-PAGE, and then assessed for (**C**) N-cadherin, vimentin, and β-Actin. Each value represents the mean ± S.E.M of three independent experiments. * *p* ≤ 0.05, ** *p* ≤ 0.01, *** *p* ≤ 0.001 compared with the correspondent control.

Furthermore, the involvement of JNK in Dox-induced cell migration was investigated using the wound healing assay, which showed that the cells treated with Dox alone were highly motile and significantly repopulated the wounded area; however, in the presence of SP600125, the migratory effect was significantly reduced (*p* ≤ 0.01) (Figure 8A). This resulted in the inhibition of the invasive ability of MDA-MB-231 BC cells after Dox treatment (Figure 8B). Invasion through the inserts increased in cells treated with Dox alone (*p* ≤ 0.01) and was remarkably reduced in the presence of the SP600125 inhibitor (*p* ≤ 0.001). These findings suggest that Dox-mediated invasion of MDA-MB-231 cells was potentiated through the JNK pathway. Consistent with its inhibitory effect on migration and invasion, SP600125 also reversed the EMT process induced by Dox treatment (Figure 8C). Pre-incubation of cells with SP600125 significantly downregulated N-cadherin (*p* ≤ 0.01) and vimentin (*p* ≤ 0.05) compared to Dox alone. Overall, the present results suggest that JNK pathway activation may contribute to Dox-induced EMT and subsequent chemoresistance. 

## 3. Discussion 

In the present study, we showed that MKP-2 is highly expressed in breast tumor tissues compared to normal adjacent tissues, which is in line with previous findings [29,30]. We also showed that MKP-2 expression is not affected by hormonal status, as its expression is high in the presence or absence of hormone receptors. This suggests that MKP-2 may not be under the control of hormonal receptors. However, the findings were not statistically significant, probably due to the small sample size. Although there is no adequate evidence to explain the role of MKP-2 in BC proliferation and progression, it has been shown that low MKP-2 expression strongly correlates with a high Ki-67 score, and patients who lost MKP-2 expression had an increased Ki-67 score after neoadjuvant chemotherapy [31]. However, the association between MKP expression and the Ki-67 index is tissue-related. The results obtained in this study showed that MKP-2 expression was not associated with Ki-67 expression, as both tissues with low and high Ki-67 staining yielded a positive stain for MKP-2 compared to adjacent normal tissues. 

Furthermore, we have shown in the present study that MKP-2 plays a role in the proliferation and chemoresistance of BC. Indeed, we have shown that MKP-2 downregulation inhibits MDA-MB-231 cell growth and enhances the cytotoxic effects of Dox. This was accompanied by MKP-2 downregulation at both the mRNA and protein level. In contrast, Dox increases the expression of MKP-2 in other cell lines, such as gastric cancer cell lines [32]. This may be due to cell (cancer) type-specific differences. Nevertheless, the obtained results are in agreement with previous findings, which showed that MKP-2 knockdown decreases the proliferation and survival of fibroblast cells [22] and BC cells [33]. This may be due to the known function of MKP-2 as a negative regulator of the MAPK pathway [34]. JNK and p38 MAPK members are regulated by MKP-2 [22,34] and are involved in the cell cycle, cell survival, and differentiation in response to chemotherapeutic agents in cancer cells [35]. Specifically, JNK activation is essential for the induction of apoptosis by a variety of stress stimuli, including ultraviolet (UV) radiation, growth factors, and chemotherapeutic drugs, and its inhibition has been linked to acquired resistance to several chemotherapeutic treatments [36,37]. In addition, recent studies have implicated JNK/c-Jun in Dox-induced apoptosis and chemoresistance [15]. In this study, Dox-dependent MKP-2 downregulation was correlated with the induction of JNK activity and upregulation of its downstream target c-Jun in a concentration- and time-dependent manner. JNK activation was positively correlated with the upregulation of apoptosis-associated proteins, such as cleaved caspases and PARP, and with DNA damage in response to Dox treatment. In addition, in the present study, Dox-induced phosphorylation of JNK/c-Jun in MAD-MB-231 cells was significantly diminished by the JNK inhibitor, SP600125, which protects cells from apoptosis and ultimately DNA damage. This indicated the contribution of JNK to Dox cytotoxicity. This is in line with previous findings that a dramatic increase in JNK phosphorylation was detected in cells isolated from MKP-2 knockout mice in response to lipopolysaccharide (LPS) [38] or anisomycin [22]. Moreover, suppression of MKP-2 expression in 293T cells demonstrated a high level of JNK phosphorylation in response to H_2_O_2_, which remained higher over six hours than that observed in control cells [39]. 

Interestingly, MKP-2 ectopic expression in non-tumorigenic BCs (MCF-12A) activated JNK, reduced Dox cytotoxicity, and significantly enhanced cell growth, confirming the role of MKP-2 in cell growth and resistance to Dox. The same observations were recorded in gastric cancer cells, where MKP-2 overexpression increased proliferation [32]. In addition, the involvement of MKP-2 overexpression in the proliferation of cancer cells has been previously established [40]. Our results also revealed that silencing MKP-2 protein enhanced cell sensitivity to Dox-induced cell cycle arrest. MKP-2 silencing induced G0/G1 BC cell cycle arrest, which was accompanied by a significant decrease in cyclin B1 and p-Cdc2 protein expression, which prevented cells from proliferating and entering the next phase. This contradicts previous findings that showed MKP-2 depletion induces a delay in the G2/M phase transition through inhibition of cyclin B1 [22,41]. Silencing MKP-2 led to a significant reduction in Dox-induced G2/M arrest, as more cells were arrested in the G0/G1 phase, resulting in significant cell death. This was accompanied by a significant decrease in cyclin B1 expression and p-Cdc2. Thus, the present findings demonstrate that MKP-2 is crucial for cell cycle regulation by controlling the G0/G1 transition in MDA-MB-231 cells. Additionally, deletion of MKP-2 may provide another therapeutic approach for BC. To the best of our knowledge, this is the first study to report this finding. In support of the importance of MKP-2 in cell growth and survival, overexpression of MKP-2 in MCF-12 cells increased cell survival and reversed Dox-induced cell apoptosis. This is in line with recent findings showing that constitutive expression of MKP-2 in cancer cells contributes to enhanced proliferation by escaping from apoptosis and cell cycle arrest [40]. Similar results were found with other MKPs, such as MKP-4, which enhanced the progression of colorectal and gastric cancers [42,43].

Additionally, MKP-2 silencing inhibited EMT by downregulating the mesenchymal markers N-cadherin, vimentin, and Snail. This was accompanied by a significant reduction in MMP levels. MMPs, which are secreted by tumor cells undergoing EMT, degrade the structural components of the extracellular matrix to enhance tumor cell migration while activating growth factors or inactivating protease inhibitors [44]. This is in line with a recent finding, which showed that MKP-2 induces EMT in gastric cancer cells and blocks EMT, successfully reversing MKP-2-mediated Dox resistance [32]. These results are also in agreement with another study that revealed that silencing of MKP-2 significantly enhanced the chemosensitivity of BC cells to Dox, while cells that expressed high levels of MKP-2 had a mesenchymal phenotype [23]. 

Hence, in the present study, we showed that MKP-2 silencing attenuated Dox-induced EMT and accelerated apoptosis by activating the JNK signalling pathway. Therefore, in the present study, we hypothesized that MKP-2 plays a major role in Dox resistance in MDA-MB-231 cells via a feedback loop involving MKP-2/JNK and the pro-metastatic EMT process.

## 4. Materials and Methods

### 4.1. Materials

Cell lines MCF-12A, MDA-MB-231, T-47D, and MCF-7 were obtained from the American Type Culture Collection (ATCC; Manassas, VA, USA) and cultured according to the manufacturer’s protocols. All cell culture materials were from Gibco (Paisley, UK). Dox hydrochloride (HCL), 3-(4,5-dimethylthiazol-2-yl)-2,5-diphenyl tetrazolium bromide, and lysis buffer were purchased from Sigma-Aldrich. The following antibodies were purchased from Cell Signalling Technology: B-cell lymphoma-extra-large (Bcl-xl), p-c-Jun, PARP, P-γ-H2AX, cleaved Caspase-3, Caspase-3, Caspase-9, Caspase-8, anti-rabbit p-JNK (Thr^183^/Tyr^185^), vimentin, Snail, and rabbit secondary antibodies. Antibodies against MKP-2, JNK, Bax, β-actin, and mouse secondary antibodies were purchased from Santa Cruz Biotechnology. Antibodies against N-cadherin were purchased from BD Biosciences. 

### 4.2. Patients Data and Tissue Specimens

The study was approved by the ethics committee of the Kuwait Ministry of Health (2017/538) and was conducted following the Ministry’s guidelines. For clinicopathologic correlation, 108 retrospective unselected formalin-fixed paraffin-embedded invasive ductal and lobular breast cancer samples were retrieved from the Adan Hospital, Histopathology Unit between 2006 and 2017. The mean age of the patients at diagnosis was 52.72 years (range, 21–88 years). The pathological parameters assessed for each patient included age at diagnosis, histopathological subtype, grade, lymph node status, immunohistochemical profile of the oestrogen (ER) and progesterone receptors (PR), and immunohistochemical profile of Ki-67 and human epidermal growth factor receptor (HER)-2 in invasive malignant cells. All haematoxylin and eosin (H&E)-stained slides, pathology reports, and other medical records were reviewed to confirm the diagnoses. After tumor size assessment, the tumors were grouped into two categories: ≤2 cm and >2 cm. Tumor grade evaluation was approved according to the established Nottingham histologic scoring system (also known as “the Elston–Ellis modification of Scarff–Bloom–Richardson grading system”), which depends on the percentage of tubular differentiation, the presence of nuclear pleomorphism, and the number of mitoses [45]. The Ki-67 proliferation index cut-off was used according to Bustreo et al. [46]. Considering a tumor ER- or PR-positive required ≥1% invasive malignant cells with nuclear staining/immunoreactivity. The HER-2 was scored according to the Herceptin test guidelines (0–3+). 

### 4.3. Immunohistochemistry (IHC)

Deparaffinized sections from paraffin blocks (4 µm thickness) were prepared for loading into a Benchmark XT Ultra machine (Ventana Inc., Harvard, MA, USA). Slides were pre-treated for antigen retrieval using the EZ prep (Ref No:950-102) and Ultra Cell Conditioner 1 (Ultra CC1—Ref No:950-224) reagents. The incubation time for antigen retrieval was standardized by validating the default protocol with different combinations of incubation times and validating the result by comparing the intensity and quality of the reaction. Different dilutions within the recommended range were prepared and run on a machine with known control slides. The results were compared, and the desired optimal dilution for MKP-2 rabbit polyclonal (1:100) antibody (Santa Cruz Biotechnologies, Dalas, USA) against dual specificity phosphatase 4 (DUSP4) and the corresponding incubation times were determined. Staining was performed using the Ventana Ultra View and Diaminobenzidine (DAB) Detection System, which includes a peroxidase inhibitor, horseradish peroxidase (HRP) multimer, DAB chromogen, hydrogen peroxide, and ultraview DAB copper. Mouse monoclonal antibodies against ER, PR, HER2/neu, and Ki-67 (Cell Marque, Rocklin, CA 95677, USA) were used according to the manufacturers’ recommendations. Slides were evaluated by a pathologist, and for each section, cells were counted in 15 fields of view using a high Olympus CX51 light microscope (400× magnification) and a double-blinded method. The percentage of positive cells was calculated as the average number of stained cells per 100 cells counted. IHC staining for MKP-2 was considered positive when brownish-yellow grains were observed in the nucleus. The scoring criteria were as follows: negative, 0 points; pale yellow, 1 point; yellow, 2 points; brownish yellow, 3 points [47]. 

### 4.4. Cell Culture

Breast cancer cells (MDA-MB-231, T-47D, and MCF-7) were grown in RPMI 1640 medium supplemented with 2 mM L-glutamine, 25 mM HEPES, 10% heat inactivated fetal calf serum, penicillin (100 unit/mL), and streptomycin (100 mg/mL) at 37 °C in a humidified atmosphere with 5% CO_2_. MCF-12A cells were cultured in a 1:1 mixture of Dulbecco’s modified Eagle’s medium and Ham’s F12 medium with 20 ng/mL human epidermal growth factor (HEGF), 100 ng/mL cholera toxin, 0.01 mg/mL bovine insulin, 500 ng/mL hydrocortisone, and bovine serum (5%). Upon reaching proper confluency (50–60%), the cells were used for virus infection, siRNA transfection, or stimulation. Cells from passages 1 to 4 were used in this study. 

### 4.5. Western Blot Analysis

After treatment, adherent cells were harvested by gently scraping the plates. The cells were then centrifuged, washed in phosphate-buffered saline (PBS), and lysed in ice-cold radioimmunoprecipitation assay (RIPA) lysis buffer containing a protease inhibitor cocktail (Roche, Mannheim, Germany) to obtain total protein. Protein concentrations were determined using an Epoch microplate spectrophotometer (gen 5 Software, Agilent, Santa Clara, CA, USA). Proteins (10–60 µg/mL) were denatured by boiling in 1× sodium dodecyl-sulphate (SDS) sample buffer with dithiothreitol (DTT) for 5 min, separated using 10–15% SDS-polyacrylamide gel electrophoresis (PAGE), and transferred to a nitrocellulose membrane. The membranes were blocked against non-specific binding for 1 h in 2% bovine serum albumin (BSA) (*w*/*v*) diluted in tris-buffered saline tween (TBST) buffer (50 mM Tris–HCl, 150 mM NaCl, 0.2% (*v*/*v*) Tween-20) and incubated overnight with the primary antibody diluted in 0.2% BSA (*w*/*v*) in TBST buffer at 4 °C. The blots were washed with TBST buffer for 5 min (3×) and incubated with an HRP-conjugated secondary antibody in 0.2% BSA (*w*/*v*) diluted in TBST buffer for 2 h. The blots were visualized using an enhanced chemiluminescence (ECL) reagent kit (Clarity Western ECL substrate, Bio-Rad). Protein detection was performed using a Bio-Rad imaging device (ChemiDoc MP; Bio-Rad Laboratories, Inc., Hercules, CA, USA). Densitometric analysis was performed using an image analysis program (ScnImage). The nitrocellulose membranes were stripped and reused for the detection of the housekeeping protein β-actin. 

### 4.6. Cell Viability Assay

Cells were seeded in 6-well plates at 3 × 10^5^ cells/well and allowed to adhere overnight. The cells were then treated and incubated for 24 h before trypsinization and counting using a Vi-Cell analyzer (Beckman Coulter, Brea, CA, USA). The machine automates the trypan blue exclusion method to assess cell viability. Data are presented as proportional viability (%) by comparing the treated group with the untreated cells, the viability of which was assumed to be 100%. Cell morphological changes were detected by either Ziess axiovert-40 phase contrast microscopy or a cell observer (10×).

### 4.7. MTT Cytotoxic Assay

Cells were seeded into 96-well plates at 2.5 × 10^4^ per well and allowed to adhere overnight. The cells were incubated with either medium or treatment (Dox, Adv-MKP-2, and MKP-2 siRNA) for 24, 48, and 72 h. The reaction was then terminated, and the medium was replaced with 100 µL of fresh medium with 10µL of 3-(4,5-dimethylthiazol-2-yl)-2,5-diphenyl tetrazolium bromide (MTT) stock solution (5 mg/1 mL) to obtain a final MTT concentration of 0.5 mg/mL in each well. After incubation for 1–2 h, the MTT solution was removed from the wells, the resulting MTT formazan was solubilized in 100 mL of dimethyl sulfoxide (DMSO), and absorbance was recorded using a multiscan spectrophotometer (Thermo Scientific, Pleasanton, CA, USA) at 570 nm with a reference wavelength of 650 nm. The effect on cell survival was assessed as the percentage of cell viability compared with that of the control cells, which were arbitrarily assigned 100% survival. 

### 4.8. Flow Cytometry

Approximately 2 × 10^4^ cells were incubated with the indicated treatment for 24 h. Subsequently, trypsin was added, and the harvested cells were washed in cold (4 °C) 1× PBS twice and centrifuged to discard the supernatant, followed by resuspension in 100 µL of 1× Binding Buffer. Next, 10 μL of Annexin V (fluorescein isothiocyanate [FITC]) and 20 μL of 7-aminoactinomycin (AAD) were added and incubated for 15 min in the dark at room temperature. The volume was made up to 500 μL with a binding buffer. Flow cytometry (Cytomic FC500, Beckman, Germany) was performed after 15 min of incubation in the dark. Data acquisition was performed using the Facsdiva 6.1 software.

### 4.9. Scratch Assay

The cells were seeded in 6-well plates and cultured for 24 h to reach 90% confluence. After scraping the cell monolayer with a sterile micropipette tip to create a wound, the wells were washed with PBS, and 1 mL of fresh medium was added. The indicated treatments were added and incubated for 24 h. The first image of each scratch was obtained at time zero. Cells that migrated into the wound surface were examined and captured under an inverted microscope (Ziess axiovert-40 phase-contrast microscopy) at the same location, and the healed area was measured. The rate of migration was determined using the following formula: (wound area at 0 h − wound area at (n) h/wound area at 0 h) × 100.

### 4.10. Transwell Invasion Assay

Cell invasion was assessed in transwell chambers equipped with 8 μM pore size and 6.5 mm diameter polycarbonated membranes (Corning Costar Inc., Corning, NY, USA). Extracellular matrix Matrigel (Corning Costar Inc., Corning, NY, USA) at 50 μL/cm^2^ was added to the surface of the polycarbonate membrane in the transwell chamber and then placed under a fume hood either at 37 °C or at room temperature for 30 min until the Matrigel was gelled. The cells were seeded into the upper wells at a concentration of 1 × 10^5^ cells/well in 100 μL serum-free medium and then cultured for 48 h following treatment. The bottom chambers of the transwell chambers were filled with 600 μL medium containing 10% fetal bovine serum (FBS). After incubation for 48 h, non-migratory cells on the upper surface of the chambers were removed using a cotton swab, and the migrated cells were fixed with 100% methanol for 10 min at room temperature and stained with 0.1% crystal violet. Five randomly chosen fields were photographed under a light microscope (Ziess axiovert-40 phase contrast microscope) at a magnification of 20×. The percentage inhibition was normalized to the percentage of untreated cells.

### 4.11. Immunofluorescent Staining 

Cells were grown in confocal dishes and, after treatment for the indicated time, fixed with 70% ice-cold methanol, and kept stable in 0.2% Triton for 10 min to rupture the cell membranes. Following 3× PBS washing, non-specific antigen binding sites were blocked with 2% BSA for 30 min. Cells were then incubated with primary antibody (1:200) for 1 h, washed with 3× PBS, and incubated for 1 h with a mixture of fluorescence-tagged secondary antibody (Alexa Fluor 555 goat anti-mouse IgG or Alexa Fluor 488 goat anti-rabbit, Thermo Fisher, USA) with nucleus staining 4′,6-diamidino-2-phenylindole (DAPI). After washing, images were captured at 40× magnification using a Zeiss LSM 510-META confocal laser-scanning microscope.

### 4.12. siRNA Transfection

MKP-2 siRNAs were purchased from Qiagen, and the target sequences were DUSP4-3 TACAGTGGATTTAGAATATAT, DUSP4-8 CTGGTTCATGGAAGCCATAGA, DUSP4-9CACTCCAACTTAGAGCAATAA, and DUSP4-10 ACCGTAGCATGCAGATGTCAA. Before transfection, 3 × 10^5^ of cells were seeded in a 12-well plate containing 0.5 mL of Opti-minimum essential media (MEM) (GIBCO, Invitrogen, Waltham, MA, USA) without serum and antibiotics. High transfection of DUSP4 siRNA was performed according to the manufacturer’s protocol to increase the transfection efficiency. To provide a baseline for comparing siRNA-treated samples, control cells were treated with negative control siRNA (Cat. No. 1022076; Qiagen, Hilden, Germany), which did not target any gene product. The required amount of siRNA (3 μL, 10 nM) was diluted in 94 μL serum-free medium, and 6 μL of HiPerFect Transfection Reagent (Qiagen, Hilden, Germany) was dissolved in the diluted siRNA, vortexed, and incubated for 15 min at room temperature. The transfection complex (103 µL) was added to the cells, gently shaken, and thoroughly mixed. The cells were incubated for 6 h at 37 °C with 5% CO_2_. Next, Opti-MEM containing 20% FBS was added to each well for a total volume of 1100 μL and incubated for 24 h. The medium was replaced with complete RPMI medium and incubated for 24 h. The cells were then trypsinized, re-transfected and incubated for 24 h. The medium was replaced with complete RPMI medium and incubated for 1 h. Then, the treatment was added, and the cells were further incubated for 24 h. Transfection efficiency was estimated by measuring MKP-2 gene knockdown using real-time quantitative reverse transcription polymerase chain reaction (qRT-PCR). To assess siRNA delivery to cells, the control siRNA FITC-Conjugate-A (Cat. No.: E1817, Santa Cruz) was used, and toxicity was estimated by comparing DAPI staining for negative siRNA-treated cells and control cells.

### 4.13. MKP-2 Adenovirus Infection 

The cells were seeded in 6/12-well plates at approximately 50% confluency and incubated for 24 h. An appropriate multiplicity of infection (MOI) of MKP-2 adenovirus (Ad-MKP-2, Cat. No. 1574, Vector Biolabs, 500 plaque forming units (pfu)/cell) was added, then incubated for 24 h at 37 °C, 5% CO_2_. The culture medium was renewed, and the cells were incubated with the indicated treatments.

### 4.14. RNA Extraction and qRT-PCR

Total RNA was extracted using TRIzol ™ reagent (Ambion, Thermo Fisher Scientific, Waltham, MA, USA) and chloroform and precipitated with isopropanol (VWR International). The precipitated RNA was washed once with 70% ethanol, air-dried, and resuspended in sterile, nuclease-free water. Then, the samples were incubated at 65 °C for 10 min and stored at −86 °C until analysis. The total RNA concentration was determined using NanoDrop, and samples were normalized to 1 mg/mL RNA. First-strand cDNA was generated using a High-Capacity cDNA Reverse Transcription kit (Applied Biosystems, Thermo Fisher Scientific, Inc., Waltham, MA, USA) according to the manufacturer’s protocol. The cDNA was used as a template for PCR amplification of the MKP-2 gene. The following primers were used for the PCR: MKP-2 forward 5′-CTCCTGTGGGACCCCACTACACGAC-3′ and reverse 5′-ATGTCTCTCTCCGGGCAGCATGGTAGG-3′; β-actin (control gene) forward 5′-GGACTTCGAGCAAGAGATGG-3′ and reverse 5′-AGCACTGTGTTGGCGTACAG-3′. Each reaction was repeated three times. All qRT-PCR analyses were performed using PowerUp SYBR Green Master Mix (Applied Biosystems, USA), and qRT-PCR reactions were conducted using a 7500 Fast Real-Time PCR system (Applied Biosystems; Thermo Fisher Scientific, Inc.) under the following conditions: 95 °C for 3 min, followed by 40 cycles of 15 s at 95 °C and 30 s at 55 °C. A negative control was used in each run to assess primer specificity and possible contamination. Real-time RT-PCR data were analyzed using the relative gene expression (ΔΔCt) method. MKP-2 gene expression data are presented as the fold change in gene expression normalized to the reference gene (β-actin). 

### 4.15. Cell Cycle

Cells were seeded, treated, and incubated for the indicated periods. The cell monolayer was harvested, washed in PBS, and fixed for 30 min in 1 mL of ice-cold 70% ethanol. The fixed cells were then pelleted, rinsed with PBS, incubated in the presence of RNase A for 15 min at 37 °C, stained with propidium iodide, and kept in the dark. The stained cells were analyzed for DNA content using a Cytomic FC500 flow cytometer (Beckman Coulter, Brea, CA, USA). Approximately 20,000 cells per sample were analyzed using excitation at 488 nm and emission at 617 nm. The percentage of cells in each phase of the cell cycle was determined using the CXP System Software (Beckman Coulter, Miami Lakes, FL, USA).

### 4.16. Statistical Analysis

Data are presented as the mean ± standard error of the mean (SEM). The Student’s *t*-test (two-tailed) was used to compare the two groups of independent samples, assuming equal variances among all experimental datasets. Pearson’s chi-squared test (χ^2^) was used to evaluate the associations between MKP-2 expression and clinicopathological factors. Additionally, correlation analysis for correlation tests was performed. *p* ≤ 0.05 was considered statistically significant in all tests. All tests were two-sided.

## 5. Conclusions 

In this study, we investigated the potential role of MKP-2 in the invasion, metastasis ability, and, subsequently, chemoresistance of MDA-231-MB in response to Dox. The results obtained indicate that MKP-2 silencing attenuated Dox-induced EMT and accelerated apoptosis by activating the JNK signalling pathway. According to the above obtained results, we hypothesized that MKP-2 plays a major role in Dox resistance in MDA-MB-231 cells via a feedback loop involving MKP-2/JNK and the pro-metastatic EMT process.

## Figures and Tables

**Table 1:** Patient’s Clinicopathological Characteristics and DUSP4 Expression.

Variable	DUSP4 Expression				χ^2^	*p*-Value
	Negative	Low	Moderate	High		
**Age groups**					8.097	0.04
**≤40 (*n* = 23)**	2 (8.7%)	10 (43.5%)	8 (34.8%)	3 (13%)		
**>40 (*n* = 85)**	29 (34.1%)	36 (42.4%)	16 (18.8%)	4 (4.7%)		
**Total 108**						
**Histologic grade**					1.2	0.977
**Grade 1 (*n* = 10)**	4 (40%)	3 (30%)	2 (20%)	1 (10%)		
**Grade 2 (*n* = 49)**	14 (28.6%)	22 (44.9%)	10 (20.4%)	3 (6.1%)		
**Grade 3 (*n* = 46)**	13 (28.3%)	19 (41.3%)	11 (23.9%)	3 (6.5%)		
**Total 105**						
**Tumor Type**					0.457	0.928
**Ductal (*n* = 76)**	30 (28.6%)	45 (42.9%)	23 (21.9%)	7 (6.7%)		
**Lobular (*n* = 3)**	1 (33.3%)	1 (33.3%)	1 (33.3%)	0		
**Total 108**						
**ER**					2.648	0.449
**Negative (*n* = 19)**	6 (31.6%)	7 (36.8%)	6 (31.6%)	0		
**Positive (*n* = 89)**	25 (28.1%)	39 (43.8%)	18 (20.2%)	7 (7.9%)		
**Total 108**						
**PR**					1.44	0.696
**Positive (*n* = 33)**	12 (36.4%)	12 (36.4%)	7 (21.2%)	2 (6.1%)		
**Negative (*n* = 75)**	19 (25.3%)	34 (45.3%)	17 (22.7%)	5 (6.7%)		
**Total 108**						
**HER-2 Score**					5.332	0.804
**Negative (*n* = 55)**	14 (25.6%)	23 (41.8%)	13 (23.5%)	5 (9.1%)		
**Equivocal (*n* = 20)**	6 (30%)	9 (45%)	4 (20%)	1 (5%)		
**Positive (*n* = 32)**	11 (34.4%)	14 (43.8%)	6 (18.8%)	1 (3.1%)		
**Total 107**						
**Ki-67 Expression**					2.535	0.469
**Low (*n* = 31)**	12 (38.7%)	13 (41.9%)	4 (12.9 %)	2 (6.5%)		
**High (*n* = 68)**	18 (26.5%)	29 (42.6%)	17 (25 %)	4 (5.9%)		
**Total 99**						
**Tumour size**					5.8	0.446
**≤2 cm** **(*n* = 9)**	4 (44.4)	2 (22.2)	2 (22.2)	1 (11.1)		
**>2 cm–5 cm (*n* = 32)**	14 (43.8)	12 (37.5)	6 (18.8)	0		
**>5 cm (*n* = 11)**	7 (63.6)	2 (18.2)	1 (9.1)	1 (9.1)		
**Total 52**						

## Data Availability

The data presented in this study are available in this article and Supplementary Materials.

## Supplementary Materials

The following supporting information can be available online https://www.mdpi.com/article/10.3390/molecules27196146/s1. Figure S1: western blots images of *p*-JNK and *p*-c-JUN (*n* = 3), Figure S2: western blots images of *p*-ERK 1/2 and *p*-P38 (*n* = 3).

## Abbreviations

DMSO	Dimethyl sulfoxide
Dox	Doxorubicin
DUSP4	Dual-specificity phosphatase 4
ECM	Extracellular matrix
EDTA	Ethylenediaminetetraacetic acid
EMT	Epithelial–mesenchymal transition
ER	Estrogen receptor
ERK1/2	Extracellular signal-regulated protein kinase
FITC	Fluorescein isothiocyanate
HER-2	Human epidermal growth factor receptor 2
IHC	Immunohistochemistry
JNK	c-Jun N-terminal kinase
KDa	Kilodalton
LPS	Lipopolysaccharides
MKP-2	Mitogen-activated protein kinase phosphatases-2
PARP	Poly-(ADP-ribose) Polymerase
PBS	Phosphate Buffered Saline
PR	progesterone receptors
SDS	Sodium Dodecyl Sulfate
siRNA	small interfering RNA
TNBC	Triple-negative breast cancer
