# Supplementary material for: DUSP4 Silencing Enhances the Sensitivity of Breast Cancer Cells to Doxorubicin through the Activation of the JNK/c-Jun Signalling Pathway
Source: Molecules. 2022 Sep 20;27(19):6146. doi: 10.3390/molecules27196146 (PMC9572343; doi:10.3390/molecules27196146)
Supplement: Supplementary file 1 [file molecules-27-06146-s001.zip › molecules-1838997-supplementary.pdf]

Supplements:

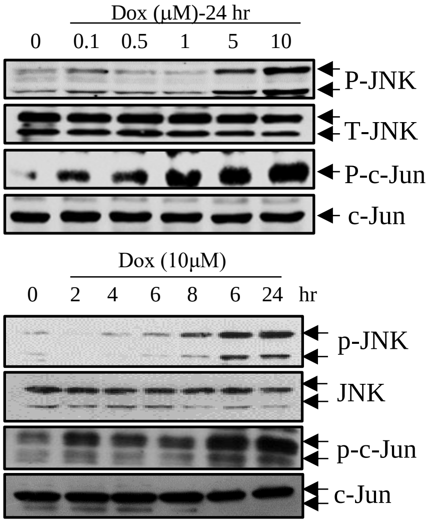

Figure S1: Doxorubicin activated JNK and its downstream protein c-Jun in a concentration- and time-dependent manner

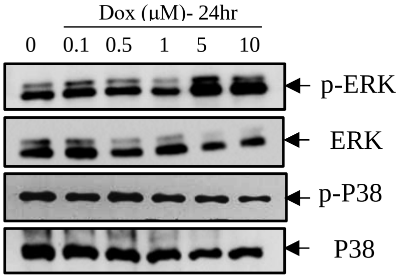

Figure S2: Effect of Doxorubicin on ERK and P38 MAPKs in MDA-MB-231 cells
